# Efficient and equitable HIV prevention: A case study of male circumcision in South Africa

**DOI:** 10.1186/1478-7547-11-1

**Published:** 2013-01-04

**Authors:** Stéphane Verguet

**Affiliations:** 1 Department of Global Health, University of Washington, 325 9th Avenue, Box 359931, Seattle, WA, 98104, USA

**Keywords:** Efficiency, Equity, HIV prevention, Male circumcision

## Abstract

**Background:**

We determine efficient, equitable and mixed efficient-equitable allocations of a male circumcision (MC) intervention reducing female to male HIV transmission in South Africa (SA), as a case study of an efficiency-equity framework for resource allocation in HIV prevention.

**Methods:**

We present a mathematical model developed with epidemiological and cost data from the nine provinces of SA. The hypothetical one-year-long MC intervention with a budget of US$ 10 million targeted adult men 15–49 years of age in SA. The intervention was evaluated according to two criteria: an efficiency criterion, which focused on maximizing the number of HIV infections averted by the intervention, and an equity criterion (defined geographically), which focused on maximizing the chance that each male adult individual had access to the intervention regardless of his province.

**Results:**

A *purely efficient* intervention would prevent 4,008 HIV infections over a year. In the meantime, a *purely equitable* intervention would avert 3,198 infections, which represents a 20% reduction in infection outcome as compared to the purely efficient scenario. A *half efficient-half equitable* scenario would prevent 3,749 infections, that is, a 6% reduction in infection outcome as compared to the purely efficient scenario.

**Conclusions:**

This paper provides a framework for resource allocation in the health sector which incorporates a simple equity metric in addition to efficiency. In the specific context of SA with a MC intervention for the prevention of HIV, incorporation of geographical equity only slightly reduces the overall efficiency of the intervention.

## Background

South Africa has the largest HIV epidemic in the world. In 2009, 5,600,000 people were living with HIV, a 18% prevalence among adults [1]. Specific features of South Africa, including a historically important migrant population, a difficult transition from apartheid that occurred at a critical juncture in the spread of the epidemic [2-4], and even after apartheid, slow government response [5] are relevant to what has been HIV’s explosive spread in the nation. A combination of domestic and international funds totaling US$ 620 million was spent on dealing with the epidemic in 2007 [1], representing a massive allocation for the national health budget.

Effective HIV prevention has eluded South Africa and sub-Saharan Africa in general. Indeed, worldwide there are few success stories: Thailand with its ‘100% condom’ intervention [6] and Uganda with its ‘ABC approach’ [7] are routinely mentioned. We need to reprioritize strategies for HIV prevention, based on evidence [8], integrated into recently scaled-up treatment [9] and tailored to the local context [10].

Consider migration patterns and lack of male circumcision (MC), which intensified the epidemic in South Africa in comparison with elsewhere. On the one hand, old patterns of worker migrations to mines persist as an inherent part of the country’s economy [3,4] and this seems unlikely to change. On the other hand, South Africa presents a relatively low rate of MC with 45% in the entire adult male population [11]. Research has shown association between lower MC rate and higher HIV prevalence in sub-Saharan Africa: a meta-analysis of observational studies found an adjusted relative risk for HIV in circumcised men of 0.42 (0.34-0.54; 95% CI) [11]. More recently, three randomized controlled trials [12-14], in South Africa, Kenya and Uganda, have consistently shown the substantial protective effect of MC upon HIV transmission from female to male. The South African trial [12] claims a 60% (32%-76%; 95% CI) protection. In the meantime, a large impact on the decrease of infections is likely with a MC intervention: Williams et al. [15] show that about 173,000 new infections per year could be prevented in South Africa with full MC coverage. In addition, the intervention would be very cost-effective [16] as compared to other HIV prevention interventions [17]. Moreover, acceptability of MC is very high: a review across studies in sub-Saharan Africa shows the median proportion of uncircumcised men willing to be circumcised to be 65% [18]. Lastly, MC provides a life-long partial protection.

In the meantime, South Africa faces unique health inequity challenges. In post-apartheid South Africa, equity naturally emerged as a top priority in social policy. Most particularly, health equity, which cuts across multiple sectors of social policy, is taken as presenting an important opportunity for rapidly achieving equity gains [19]. Civil society has played a preeminent role in making health equity a quintessential component of the country’s social agenda [19]. For example, fighting against the obstacles faced by HIV positive people in getting access to antiretroviral therapies, the Treatment Action Campaign founded in 1998 has been advocating for “a unified quality health care system which provides equal access to HIV prevention and treatment services for all people” [20].

In this paper, we model the impact over one year of a US$10 million MC intervention in South Africa, targeting adult men 15–49 years of age. Our case study includes all nine South African provinces: Western Cape, Eastern Cape, Northern Cape, Free State, KwaZulu-Natal, North West, Gauteng, Mpumalanga, and Limpopo. The goal is to examine efficiency-equity tradeoffs in resource allocation. We evaluate the intervention along two criteria: an efficiency criterion, which quantifies the number of HIV infections averted, and an equity criterion, which quantifies the likelihood of access that each adult male individual has to MC, regardless of his province. The use of a geographically defined equity criterion, as one form of vertical equity within the health sector, does not intend to capture a thorough description of equity, which is beyond the scope of this work. Geographical equity was selected in consideration of ease of exposition and data availability, and because it captures two (of three) principles of equity recently described by Jones [21]: equal life chances, or the fact that there should be no differences in outcome based on factors for which people cannot be held responsible, and equal concern for people’s needs, or the fact that some goods/services are matters of necessity and should be distributed in proportion to people’s level of need and nothing else, the government being responsible for providing equal access to health care in all parts of the country. In addition, addressing the substantial disparities in health care resources between provinces is regarded as the South African health sector’s major challenge [19].

There are multiple criteria involved in the kind of public decision making that occurs in the prioritization of health interventions [22]. The trade-offs between efficiency and equity are among these criteria, and have long been emphasized in the field of HIV/AIDS treatment and prevention [23,24]. Several mathematical frameworks, including mathematical programming, have been proposed to incorporate equity considerations into resource allocation in the public sector [25-29]. Several of these models and tools have been applied to paradigmatic HIV/AIDS policy examples [30-34]. Our goal in this paper is to present a simple mathematical model which assesses the impact of health interventions according to two comparable dimensions of efficiency and equity. MC is analyzed through the mathematical framework to indicate possibilities for resource allocation in HIV prevention, with the goal of providing insight and valuable guidance in the design of such health interventions.

## Methods

### Intervention costs

We use the data and methodology of Auvert et al. [35]. The levels of MC coverage will be low, and we therefore assume the intervention uses the existing public infrastructures and services for HIV prevention and treatment, which consist of district-level hospitals in each province. For this reason, we do not include additional infrastructure building or development costs. Trained medical practitioners will perform the operation, and also respond to potential adverse events during surgery. Costs include fixed costs and functional costs. Fixed costs include medical equipment and certification of trained circumcisers. Functional costs, which are variable, include oversight and promotion (management, monitoring, communication and advertising), salaries of full-time medical practitioners, surgical staff and counselors (for each medical practitioner, we allocate 1 medical assistant and ½ counselor). The functional costs also include surgical supplies (drugs, anesthesia and instruments), facility and program overhead (administration, facility operating costs, maintenance). All costs are in 2007 US$ and corresponding inputs are listed in Table 1. The cost function per facility *i* is given by:

(1) $$F_{i}=\alpha C_{F}+N_{i}\left(C_{T}+\beta \left[\gamma C_{c}+pd^{C}C_{s}\right]\right)$$

where *N*_*i*_ is the number of circumcisers per facility, *d* is the number of days worked by a circumciser in the year, *p*_*C*_ is the number of MCs realized by a circumciser per day. *C*_*F*_, *C*_*T*_, *C*_*c*_, and *C*_*s*_ are respectively the initial investment per circumcision facility, the initial training cost per circumciser, the salary of each circumciser and the supplies cost per patient circumcised. *α* = 1.26, *β =*1.67 and *γ =*12*1.59, are extracted from Auvert and colleagues [35] (Table 1).

**Table 1:** Cost parameters for the male circumcision intervention

** Intervention**	** Costs (2007 US$)**
Initial investment per circumcision facility C_F_	28,778
Initial training per circumciser *C*_*T*_	8,985
Salary of each circumciser *C*_*C*_	2,246
Salary of health care workers/counselors per circumciser	59% of circumciser’s *C*_*C*_
Cost of supplies per patient circumcised *C*_*S*_	11
Facility overhead costs	67% of direct salary and supply costs
Oversight and promotion costs	26% of facility-level costs
Number of MC realized per day per circumciser *p*_*C*_	10
Number of days worked by a circumciser in a year *d*	235^*^

*235 = 365 days (1 year) – 52 weeks*2 (weekends) – 26 days (holidays).

MC, male circumcision.

Source: Auvert et al. (2008) [35].

The impact of the MC intervention is assessed according to two comparable criteria: an epidemiologic efficiency criterion and an equity criterion.

### Efficiency criterion

The nine South African provinces show differing degrees of severity in the presentation of the epidemic [36] (Table 2). KwaZulu-Natal is the most severely affected, with an adult HIV prevalence of 25.8%, and the Western Cape is the least affected, with an adult HIV prevalence of 5.3% [36].

**Table 2:** Demographic, epidemiologic and behavioral features for the nine South African provinces

** Province**	Male adult population*	**Adult HIV prevalence (%)**	**Condom use (%)**	Adult men** circumcised (%)
Eastern Cape	1,294,014	15.2	70.0	43.8
Northern Cape	214,101	9.0	52.6	34.1
Western Cape	1,079,799	5.3	49.0	67.5
Free State	725,409	18.5	64.8	70.7
Gauteng	2,338,685	15.2	57.6	25.2
KwaZulu-Natal	1,994,776	25.8	66.2	26.8
Limpopo	986,827	13.7	68.0	47.5
Mpumalanga	708,304	23.1	70.2	36.3
North West	882,147	17.7	62.0	32.8

* Adapted from [37] with an annual population growth rate of 2% since 1996.

** 15–59 years of age.

Source: Shisana et al. (2009) [36]; South Africa Demographic and Health Survey 2003 [38].

We look at the effect over a year of the MC intervention on the male population, by calculating the risk of a man *R*_*m*_ getting infected by his female partner. This allows us to use a static model of transmission. The model does not look at the effect on the female population as there is not yet full evidence of a change in HIV transmission from male to female with MC [39]. The model excludes substantial longer-term potential benefits, including secondary infections in women. Our interest is in quantifying the change due to MC in HIV infections in each province population *P*. The risk for a man *R*_*m*_ to get infected by a female partner over a month [40] is:

(2) $$\begin{array}{ll} R_{m}=H_{w}[1- & S_{m}{\left(1-p\left(1-f_{\mathrm{MC}}\right)\left(1-f\right)\right)}^{\text{NB}}{\left(1-p\left(1-f\right)\right)}^{N\left(1-B\right)}\\ & -\left(1-S_{m}\right){\left(1-p\left(1-f\right)\right)}^{\mathrm{NB}}{\left(1-p\right)}^{N\left(1-B\right)}] \end{array}$$

where *H*_*w*_ is the HIV prevalence among adult women, *S*_*m*_ is the proportion of men circumcised, *p* is the probability of HIV transmission from female to male, *f* is the condom effectiveness, *f*_*MC*_ is the MC effectiveness, *N* is the number of sexual episodes in a month and *B* is the proportion of those protected by condoms. After one year, the change in new infections in a given population *P* is about:

(3) $$\Delta I_{m}=12H_{w}\mathrm{NP}\left[1-H_{m}\right]\left[B\left(1-f\right)+1-B\right]f_{\mathrm{MC}}\Delta S_{m}$$

where *H*_*m*_ is HIV prevalence among adult men, *ΔS*_*m*_ is the proportion of men circumcised due to the intervention. Table 3 lists the input values used in the analysis. The most efficient allocation is the one that maximizes *E*_*ef*_ given by:

(4) $$E_{\mathrm{ef}}=\sum_{i=1}^{9}12H_{w_{i}}NP_{i}\left[1-H_{m_{i}}\right]\left[B_{i}\left(1-f\right)+1-B_{i}\right]f_{\mathrm{MC}}\Delta S_{m_{i}}$$

where *i* refers to a province, and we assume that parameter *N* does not vary from one province to another.

**Table 3:** Parameter inputs and corresponding references

** Input**	**Value**	**Reference**
Number of female partners per man	1	Assumed
Probability of female to uncircumcised male HIV transmission	0.0038	[41]
Number of sexual acts per month	9.8	[42]
Effectiveness of condoms	0.95	[43]
Effectiveness of male circumcision	0.60	[12]

### Equity criterion

In addition to epidemiologic benefits, our goal here is to include an equity criterion in the allocation of resources. We use geographical distribution as a measure of equity at the province level in South Africa. In other words, adult men, regardless of the province they are from, should have equal chance of having access to MC surgery. This statement can be mathematically translated by the minimization of an equity objective function. An equitable allocation will seek to minimize the following equity objective function [44]:

(5) $$E_{\mathrm{eq}}=\sum_{i=1}^{9}{\left(\frac{S_{i}}{P_{i}}-\frac{S}{P}\right)}^{2}$$

where *S*_*i*_ and *P*_*i*_ are respectively the number of circumcisions realized and the population in province *i*. *S* and *P* are respectively the total number of circumcisions realized and the total population in the country: $S=\sum_{i=1}^{9}S_{i},P=\sum_{i=1}^{9}P_{i}$

### Efficiency and equity combined objective function

We now combine efficiency and equity, giving them the same weight, and need to maximize the following objective function:

(6) $$G=E_{\mathrm{ef}}/E_{ef_{\max}}+1-E_{\mathrm{eq}}/E_{eq_{\max}}$$

where *E*_*efmax*_ and *E*_*eqmax*_ are respectively the optima achieved in efficiency (4) and equity (5). An alternative way to combine efficiency and equity is to allow different weights for each of them, to reflect the respective importance one gives to them in the decision-making process. We now obtain:

(7) $$G=w_{\mathrm{ef}}E_{\mathrm{ef}}/E_{ef_{\max}}+w_{\mathrm{eq}}\left(1-E_{\mathrm{eq}}/E_{eq_{\max}}\right)$$

## Results

**Figure 1:**
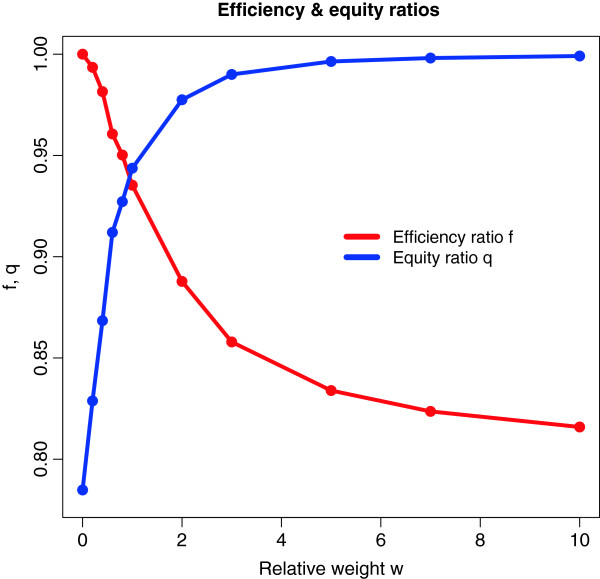
**Efficiency ratio *f = E***_***ef***_***/E***_***efmax***_**and equity ratio *q = 1-E***_***eq***_***/E***_***eqmax***_**as a function of relative weight *w = w***_***eq***_***/w***_***ef***_ .

Optimization calculations were realized using Mathematica (Wolfram Research, Inc., Mathematica, Version 8.0, Champaign, IL (2010)). In Tables 4 and 5 we report the results for the allocation of circumcisers per province, the number of circumcisions realized, and the final number of HIV infections averted over a year among the different provinces. Results are provided for three scenarios: 1. “Purely efficient allocation” (maximization of (4)), 2. “purely equitable allocation” (minimization of (5)), and 3. “half efficient/half equitable allocation” (maximization of (6)). Results for the total number of HIV infections averted over a year according to scenarios with different weight functions for the efficiency and equity criteria as defined in (7) are presented in Table 5. Figure 1 presents the evolution of the ratios *E*_*ef*_*/E*_*efmax*_ and *1-E*_*eq*_*/E*_*eqmax*_ as a function of *w*_*eq*_*/w*_*ef*_.

**Table 4:** Allocation of circumcisers and circumcisions among each province for the different scenarios

	**Scenario 1**		**Scenario 2**		**Scenario 3**	
** Province**	**Circumcisers allocated**	**Circumcisions realized**	**Circumcisers allocated**	**Circumcisions realized**	**Circumcisers allocated**	**Circumcisions realized**
Eastern Cape	0	0	8	18,800	13	30,550
Northern Cape	0	0	2	4,700	1	2,350
Western Cape	0	0	5	11,750	0	0
Free State	0	0	3	7,050	2	4,700
Gauteng	0	0	21	49,350	4	9,400
KwaZulu-Natal	75	176,250	16	37,600	42	98,700
Limpopo	0	0	6	14,100	0	0
Mpumalanga	0	0	7	16,450	7	16,450
North West	0	0	7	16,450	6	14,100
South Africa	75	176,250	75	176,250	75	176,250

Scenarios: (1) ‘Purely efficient allocation’; (2) ‘purely equitable allocation’; (3) ‘half efficient/half equitable allocation’.

**Table 5:** **Number of HIV infections averted for different efficient/equitable scenarios with the relative weight *w***_***eq***_***/w***_***ef***_

**Relative weight *w***_***eq***_***/w***_***ef***_	**Number of HIV infections averted**
0 ^a^	4,008
0.5	3,889
1 ^b^	3,749
2	3,559
3	3,438
4	3,378
5	3,342
10	3,270
∞ ^c^	3,198

^a^ ‘Purely efficient allocation’; ^b^ ‘half efficient/half equitable allocation’; ^c^ ‘purely equitable allocation’.

The number of circumcisers and the number of men circumcised will be respectively 75 and 176,250 in each scenario (Table 4). Using only the efficiency criterion to allocate the number of circumcisers per facility leads to placing all circumcisers in the province with the highest prevalence of HIV i.e. KwaZulu-Natal (Table 4) and will avert 4,008 HIV infections (Table 5). Using only the equity criterion to allocate the number of circumcisers per facility leads to placing circumcisers in all provinces (Table 4) and will prevent 3,198 HIV infections (Table 5)*.* Lastly, giving an equal weight to efficiency and equity criteria in the allocation of circumcisers per facility leads to placing circumcisers in seven provinces, excluding the Western Cape and Limpopo (Table 4). This will allow the aversion of 3,749 HIV infections (Table 5)*.* In the meantime, Figure 1 shows that in the case of a purely efficient scenario (*w*_*eq*_ *= 0*), the intervention reaches about 78% of its equity potential, whereas when the scenario moves to a high equity level (*w*_*eq*_*/w*_*ef*_ *= 10*), the intervention reaches about 82% of its efficiency potential. When the scenario is half efficient/half equitable (*w*_*eq*_*/w*_*ef*_ *= 1*), the intervention reaches about 94% of its efficiency potential.

## Discussion

We proposed here a MC intervention with a US$ 10 million budget, targeting adult men aged 15 to 49 years of age across the 9 provinces of South Africa. The intervention was delivered *efficiently*, *equitably* (following a definition of geographical equity), or with a trade-off between efficiency and equity at the provincial level. Specifically, we showed that: a *purely efficient* intervention would avert 4,008 HIV infections over a year; a *purely equitable* intervention would otherwise avert 3,198 HIV infections, which represents a 20% reduction in the health outcome as compared to the purely efficient scenario. A *half efficient-half equitable* scenario would avert 3,749 HIV infections, which is a 6% reduction in the health outcome as compared to the purely efficient scenario. Therefore, in the specific context of this MC intervention in South Africa, incorporating a geographical equity metric into the resource allocation process does not substantially negatively affect the overall efficiency of the intervention.

The deterministic epidemiologic model used in this analysis presents some limitations. First, the model does not incorporate concurrency and multiple partnerships, when on average 11% of South Africans report having had more than one sexual partner in the last 12 months [36]. Migration patterns also have the potential to intensify the effects of the intervention, to the extent that it reaches men who migrate who have concurrent and/or multiple partnerships. A second limitation is that it does not incorporate potential risk compensation in newly circumcised men with, for example, a potential reduction in condom use or a potential increase in the number of sex partners [45]. A third limitation is methodological: the model assumes homogenous mixing and does not take into account secondary HIV infections. In addition, the model is static and looks at a one-year intervention only, which is a very short period of time given the timescale of HIV infection and HIV epidemic spread. Lastly, conceptually, it does not take into account potential barriers to the uptake of MC, such as the issue of cultural acceptability.

More importantly, the framework used focused only on one specific definition of equity i.e. geographical equity. There are many other dimensions of equity such as those in Jones’ three principles [21]: 1. Equal life chances; 2. equal concern for people’s needs; 3. meritocracy: positions in society and rewards should be distributed to reflect differences in effort and ability. In looking at resource allocation geographically, the framework also only captures some aspects of vertical equity. It does not identify specific vulnerable groups (say clients of female sex workers, which could merit more weight in the allocation of resources), mechanisms improving resource distribution, or access to care within the provinces [19]. Additional mathematical modeling could easily capture those dimensions as well as look at quantifying and incorporating the urban–rural divide [46] or the physical access to clinical services using geographical information systems [47] into the resource allocation problem. Using only an efficiency criterion results in all circumcisers being allocated to KwaZulu-Natal province. That course of action, or the option provided for giving equal weight to equity and efficiency–excluding two provinces–may or may not be politically and ethically acceptable. However, the importance of the result from this model is that including equity reduces efficiency very little in this case study; the distribution of equity itself could in fact be implemented in many different ways.

This paper focuses on introducing a framework. The purpose is not exhaustive mathematical/computational modeling of the MC intervention, nor a detailed definition of equity. Our goal is to present a simple model with mathematical elements which can provide insight into resource allocation. As the MC case study shows, it can help guide the design of health interventions. Despite context-specific results – depending on the levels and distribution of HIV burden in the country (South Africa here), and the definition of equity (geographical equity here) – this paper presents a generalizable, intuitive way to incorporate an equity metric into resource allocation for health interventions, while allowing for a calculation of how such incorporation affects efficiency. We hope to export the methodology to other settings/countries, and adapt the model for other preventive and curative interventions, for a various array of diseases and conditions.

## Conclusions

This paper proposed a framework of resource allocation which incorporates a simple equity metric in addition to efficiency. The mathematical model developed was used to assess the impact of a health intervention according to two comparable dimensions of efficiency and equity, and has the potential to provide valuable guidance in the design of such health interventions. In the specific example of a male circumcision intervention for the prevention of HIV in South Africa, the incorporation of geographical equity into the resource allocation did not substantially diminish the maximal efficiency of the intervention. The importance of the model is that it allows a simple measure of equity to be included in what are usually purely efficiency calculations.

## Competing interests

The author declares that he has no competing interests.

## References

[B1] UNAIDS South Africa Accessed December 26, 2012 at http://www.unaids.org/en/regionscountries/countries/southafrica/

[B2] Abdool Karim Q South Africa: host to a new and emerging HIV epidemic Sex Transm Infect 1999 75 139 147 10.1136/sti.75.3.139 10448384 PMC1758212

[B3] Lurie MN Williams BG Zuma K Mkaya-Mwamburi D Garnett GP Sturm A Sweat M Gittelsohn J Abdool Karim SS The impact of migration on HIV-1 transmission in South Africa Sex Transm Dis 2003 30 2 149 156 10.1097/00007435-200302000-00011 12567174

[B4] Hargrove JW Migration, mines and mores: the HIV epidemic in Southern Africa S Afr J Sci 2008 104 53 61

[B5] Chigwedere P Seage GR III Gruskin S Lee TH Essex M Estimating the lost benefits of antiretroviral drug use in South Africa AIDS 2008 49 4 410 415 10.1097/qai.0b013e31818a6cd5 19186354

[B6] Celentano DD Nelson KE Lyles CM Beyrer C Eiumtrakul S Go V Kuntolbutra S Khamboonruang C Decreasing incidence of HIV and sexually transmitted diseases in young Thai men: evidence for success of the HIV/AIDS control and prevention program AIDS 1998 12 F29 F36 10.1097/00002030-199805000-00004 9543437

[B7] Green EC Halperin DT Nantulya V Hogle JA Uganda’s HIV prevention success: the role of sexual behavior change AIDS Behav 2006 10 4 335 346 10.1007/s10461-006-9073-y 16688475 PMC1544373

[B8] Potts M Halperin DT Kirby D Swidler A Marseille E Klausner JD Hearst N Wamai RG Kahn JG Walsh J Reassessing HIV prevention Science 2008 320 749 750 10.1126/science.1153843 18467575 PMC3501984

[B9] Piot P AIDS: from crisis management to sustained strategic response Lancet 2006 368 S26 30 10.1016/S0140-6736(06)69161-7 16890840

[B10] Wegbreit J Bertozzi S DeMaria LM Padian N Effectiveness of HIV prevention strategies in resource-poor countries: tailoring the intervention to the context AIDS 2006 20 9 1217 1235 10.1097/01.aids.0000232229.96134.56 16816550

[B11] Weiss HA Quigley MA Hayes RJ Male circumcision and risk of HIV infection in Sub-Saharan Africa: a systematic review and meta-analysis AIDS 2000 14 2361 2370 10.1097/00002030-200010200-00018 11089625

[B12] Auvert B Taljaard D Lagarde E Sobngwi-Tambekou J Sitta R Puren A Randomized, controlled intervention trial of male circumcision for reduction of HIV infection risk: The ANRS 1265 Trial PLoS Med 2005 2 11 e298 10.1371/journal.pmed.0020298 16231970 PMC1262556

[B13] Bailey RC Moses S Parker CB Agot K Maclean I Krieger JN Williams CFM Campbell RT Ndinya-Achola JO Male circumcision for HIV prevention in young men in Kisumu, Kenya: a randomized controlled trial Lancet 2007 369 643 656 10.1016/S0140-6736(07)60312-2 17321310

[B14] Gray RH Kigozi G Serwadda D Makumbi F Watya S Nalugoda F Kiwanuka N Moulton LH Chaudhary MA Chen MZ Sewankambo NK Wabwire-Mangen F Bacon MC Williams CFM Opendi P Reynolds SJ Laeyendecker O Quinn TC Wawer M Male circumcision for HIV prevention in men in Rakai, Uganda: a randomized trial Lancet 2007 369 657 666 10.1016/S0140-6736(07)60313-4 17321311

[B15] Williams BG Lloyd-Smith JO Gouws E Hankins C Getz W Hargorve J de Zoysa I Dye C Auvert B The potential impact of male circumcision on HIV in Sub-Saharan Africa PLoS Med 2006 3 7 e262 10.1371/journal.pmed.0030262 16822094 PMC1489185

[B16] Kahn JG Marseille E Auvert B Cost-effectiveness of male circumcision for HIV prevention in a South African setting PLoS Med 2006 3 12 e7 17194197 10.1371/journal.pmed.0030517 PMC1716193

[B17] Bertozzi S Padian NS Wegbreit J DeMaria LM Feldman B Gayle H Gold J Grant R Isbell MT Jamison DT, Breman JG, Measham AR, Alleyne G, Clamson M, Evans DB, Jha P, Mills A, Musgrove P HIV/AIDS prevention and treatment Disease Control Priorities in Developing Countries 2006 The World Bank Group, Washington DC

[B18] Westercamp N Bailey RC Acceptability of male circumcision for prevention of HIV/AIDS in Sub-Saharan Africa: a review AIDS Behav 2007 11 341 355 10.1007/s10461-006-9169-4 17053855 PMC1847541

[B19] McIntyre D Gilson L Putting equity in health back onto the social policy agenda: experience from South Africa Soc Sci Med 2002 54 1637 1656 10.1016/S0277-9536(01)00332-X 12113446

[B20] Treatment Action Campaign About the Treatment Action Campaign Accessed December 26, 2012 at http://www.tac.org.za/community/about

[B21] Jones H Equity in development. Why is it important and how to achieve it. Working paper 311 2009 Overseas Development Institute, London Accessed December 26, 2012, at http://www.odi.org.uk/resources/docs/4577.pdf

[B22] Baltussen R Niessen L Priority setting of health interventions: the need for multi-criteria decision analysis Cost Effective Resour Allocat 2006 4 14 10.1186/1478-7547-4-14 PMC1560167 16923181

[B23] Kaplan EH Merson MH Allocating HIV-prevention resources: balancing efficiency and equity Am J Public Health 2002 92 12 1905 1907 10.2105/AJPH.92.12.1905 12453805 PMC1447350

[B24] Cleary S Equity and efficiency in scaling up access to HIV-related interventions in resource-limited settings Curr Opin HIV AIDS 5 210 214 20539076 10.1097/COH.0b013e3283384a6f

[B25] Segall RS Some nonlinear optimization modeling for planning objectives of large market-oriented systems: with an application to real health data Appl Math Model 1989 13 203 214 10.1016/0307-904X(89)90078-4

[B26] Birch S Gafni A Cost-effectiveness/utility analyses. Do current decisions lead us to where we want to be? J Health Econ 1992 11 279 296 10.1016/0167-6296(92)90004-K 10122540

[B27] Stinnett AA Paltiel DA Mathematical programming for the efficient allocation of health care resources J Health Econ 1996 15 641 653 10.1016/S0167-6296(96)00493-6 10164046

[B28] Bleichrodt H Diecidue E Quiggin J Equity weights in the allocation of health care: the rank-dependent QALY model J Health Econ 2004 23 157 171 10.1016/j.jhealeco.2003.08.002 15154692

[B29] Epstein D Chalabi Z Claxton K Sculpher M Efficiency, Equity, and Budgetary Policies: Informing Decisions Using Mathematical Programming Med Decis Mak 2007 27 128 137 10.1177/0272989X06297396 17409363

[B30] Earnshaw SR Hicks K Richter A Honeycutt A A linear programming model for allocating HIV prevention funds with state agencies: a pilot study Health Care Manag Sci 2007 10 239 252 10.1007/s10729-007-9017-8 17695135

[B31] Lasry A Zaric GS Carter MW Multi-level resource allocation for HIV prevention: A model for developing countries Eur J Oper Res 2007 18 786 799

[B32] Richter A Hicks KA Earnshaw SR Honeycutt AA Allocating HIV prevention resources: A tool for state and local decision making Health Policy 2008 87 342 349 10.1016/j.healthpol.2008.01.008 18342388

[B33] Lasry A Carter MW Zaric GS S4HARA: System for HIV/AIDS resource allocation Cost Effective Resour Allocat 2008 6 7 10.1186/1478-7547-6-7 PMC2386442 18366800

[B34] Cleary S Mooney G McIntyre D Equity and Efficiency in HIV-treatment in South Africa: The contribution of mathematical programming to priority setting Health Econ 2010 19 10 1166 1180 10.1002/hec.1542 19725025

[B35] Auvert B Marseille E Korenromp EL Lloyd-Smith J Sitta R Taljaard D Pretorius C Williams B Kahn JG Estimating the resources needed and savings anticipated from roll-out of adult male circumcision in Sub-Saharan Africa PLoS One 2008 3 8 e2679 10.1371/journal.pone.0002679 18682725 PMC2475667

[B36] Shisana O Rehle T Simbayi LC Zuma K Jooste S Pillay-van-Wyk V Mbelle N Van Zyl J Parker W Zungu NP Pezi S & the SABSSM III Implementation Team South African national HIV prevalence, incidence, behaviour, and communication survey 2008: a turning tide among teenagers? 2009 HSRC Press, Cape Town

[B37] Statistics South Africa The people of South Africa: population census 1996, census in brief/Statistics South Africa 1998 Statistics South Africa, Pretoria

[B38] Department of Health, ORC Macro South Africa Demographic and Health Survey 2003: preliminary report 2004 Department of Health, Pretoria

[B39] Weiss HA Hankins CA Dickson K Male circumcision and risk of HIV infection in women: a systematic review and meta-analysis Lancet 2009 9 669 677 10.1016/S1473-3099(09)70235-X 19850225

[B40] Pinkerton SD Abramson PR The Bernoulli-process of HIV transmission: applications and implications 1998 Plenum Press, New York, In Handbook of economic evaluation of HIV prevention programs Edited by Holtgrave DR

[B41] Boily MC Baggaley RF Wang L Masse B White RG Hayes RJ Alary M Heterosexual risk of HIV-1 infection per sexual act: systematic review and meta-analysis of observational studies Lancet Infect Dis 2009 9 118 129 10.1016/S1473-3099(09)70021-0 19179227 PMC4467783

[B42] Wawer M Gray RH Sewankambo NK Serwadda D Li X Laeyendecker O Kiwanuka N Kigozi G Kiddugavu M Lutalo T Nalugoda F Wabwire-Mangen F Meehan MP Quinn TC Rates of HIV-1 transmission per coital act, by stage of HIV-1 infection, in Rakai, Uganda J Infect Dis 2005 191 1403 1409 10.1086/429411 15809897

[B43] Pinkerton SD Abramson PR Effectiveness of condoms in preventing HIV transmission Soc Sci Med 1997 44 9 1003 1012 9141163 10.1016/s0277-9536(96)00258-4

[B44] Wilson DP Blower SM Designing equitable antiretroviral allocation strategies in resource-constrained countries PLoS Med 2005 2 2 e50 10.1371/journal.pmed.0020050 15737005 PMC549597

[B45] Kalichman S Eaton L Pinkerton S Circumcision for HIV prevention: failure to fully account for behavioral risk compensation PLoS Med 2007 4 3 e138 10.1371/journal.pmed.0040138 17388676 PMC1831748

[B46] Wilson DP Kahn J Blower SM Predicting the epidemiological impact of antiretroviral allocation strategies in KwaZulu-Natal: The effect of the urban–rural divide Proc Natl Acad Sci 2006 103 38 14228 14233 10.1073/pnas.0509689103 16968786 PMC1599939

[B47] Noor AM Zurovac D Hay SI Ochola SA Snow RW Defining equity in physical access to clinical services using geographical information systems as part of malaria planning and monitoring in Kenya Trop Med Int Health 2003 8 10 917 926 10.1046/j.1365-3156.2003.01112.x 14516303 PMC2912492

